# Force‐Based Wetting Characterization of Stochastic Superhydrophobic Coatings at Nanonewton Sensitivity

**DOI:** 10.1002/adma.202105130

**Published:** 2021-09-01

**Authors:** Matti J. Hokkanen, Matilda Backholm, Maja Vuckovac, Quan Zhou, Robin H. A. Ras

**Affiliations:** ^1^ Department of Applied Physics Aalto University School of Science Puumiehenkuja 2, 02150 Espoo, P.O. Box 15100 Aalto FI‐00076 Finland; ^2^ Department of Electrical Engineering and Automation Aalto University School of Electrical Engineering P.O. Box 15500 Aalto FI‐00076 Finland; ^3^ Department of Bioproducts and Biosystems Aalto University School of Chemical Engineering P.O. Box 16000 Aalto FI‐00076 Finland

**Keywords:** contact angle, droplet adhesion, superhydrophobicity, wetting

## Abstract

Superhydrophobic coatings have extraordinary properties like self‐cleaning and staying dry, and have recently appeared on industrial and consumer markets. The stochastic nature of the coating components and coating processes (e.g., spraying, painting) affects the uniformity of the water repellency across the coated substrate. The wetting properties of those coatings are typically quantified on macroscale using contact angle goniometry (CAG). Here, highly sensitive force‐based methods, scanning droplet adhesion microscopy (SDAM), and micropipette force sensor (MFS), are used, to quantify the microscale heterogeneity in the wetting properties of stochastic superhydrophobic coatings with irregular surface topography that cannot be investigated by CAG. By mapping the wetting adhesion forces with SDAM and friction forces with MFS, it is demonstrated that even the best coatings on the market are prone to heterogeneities that induce stick–slip motion of droplets. Thus, owing to their high spatial and force resolution, the advantages of these techniques over CAG are demonstrated.

## Graphical abstract

Scanning droplet adhesion microscopy and micropipette‐based droplet friction measurements are used for quantitative evaluation of the wetting properties of stochastic superhydrophobic coatings. These novel force‐based characterization techniques provide a detailed picture of the wetting performance and heterogeneity of super‐repellent surfaces, far beyond what is afforded by traditional contact angle measurements.

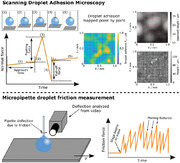

## 1 Introduction

Liquid‐repellent surfaces are of great interest in contemporary materials science due to their numerous applications, such as, self‐cleaning,^[^ ^1^, ^2^ ^]^ anti‐icing,^[^ ^3^, ^4^, ^5^ ^]^ and anti‐fogging,^[^ ^6^, ^7^ ^]^ as well as, their striking fluid dynamics properties.^[^ ^8^, ^9^, ^10^, ^11^, ^12^ ^]^ Among the different types of liquid‐repellent surfaces, superhydrophobic materials, and coatings are well‐established. Effective superhydrophobic surfaces must possess both topographic roughness and water‐repellent surface chemistry. Micro‐ or nanopillar surfaces, fabricated via micropatterning followed by surface‐chemical modification^[^ ^13^, ^14^ ^]^ are frequently used to systematically explore superhydrophobic properties. However, their fabrication requires lithographic processes that are not economically viable for large‐scale applications.^[^ ^15^ ^]^ To overcome this problem, numerous alternative solutions for preparing superhydrophobic surfaces and materials have been reported.^[^ ^16^, ^17^ ^]^ Among these, coatings deposited by spraying have found markets in both industrial and consumer applications.^[^ ^15^ ^]^ Yet, the stochastic nature of the spray deposition processes and materials results in variations of coating uniformity, and poses challenges for delivering a consistent high coating quality. The systematic quantitative evaluation of the wetting performance of these widely used coatings is largely lacking,^[^ ^18^ ^]^ and could benefit optimization of coating procedures and coating formulations.

Wetting characterization of surfaces has traditionally been done via optical contact angle goniometry (CAG).^[^ ^19^ ^]^ This technique faces reduced accuracy on highly non‐wetting surfaces such as superhydrophobic coatings, where the error in advancing and receding contact angles can be up to 10°.^[^ ^20^, ^21^, ^22^ ^]^ Furthermore, it is commonly understood that these measurements are not suitable for studying the spatial heterogeneity of surface wettability since the lateral resolution of several millimeters results in averaged wetting properties over large areas.^[^ ^23^ ^]^

**Figure 1:**
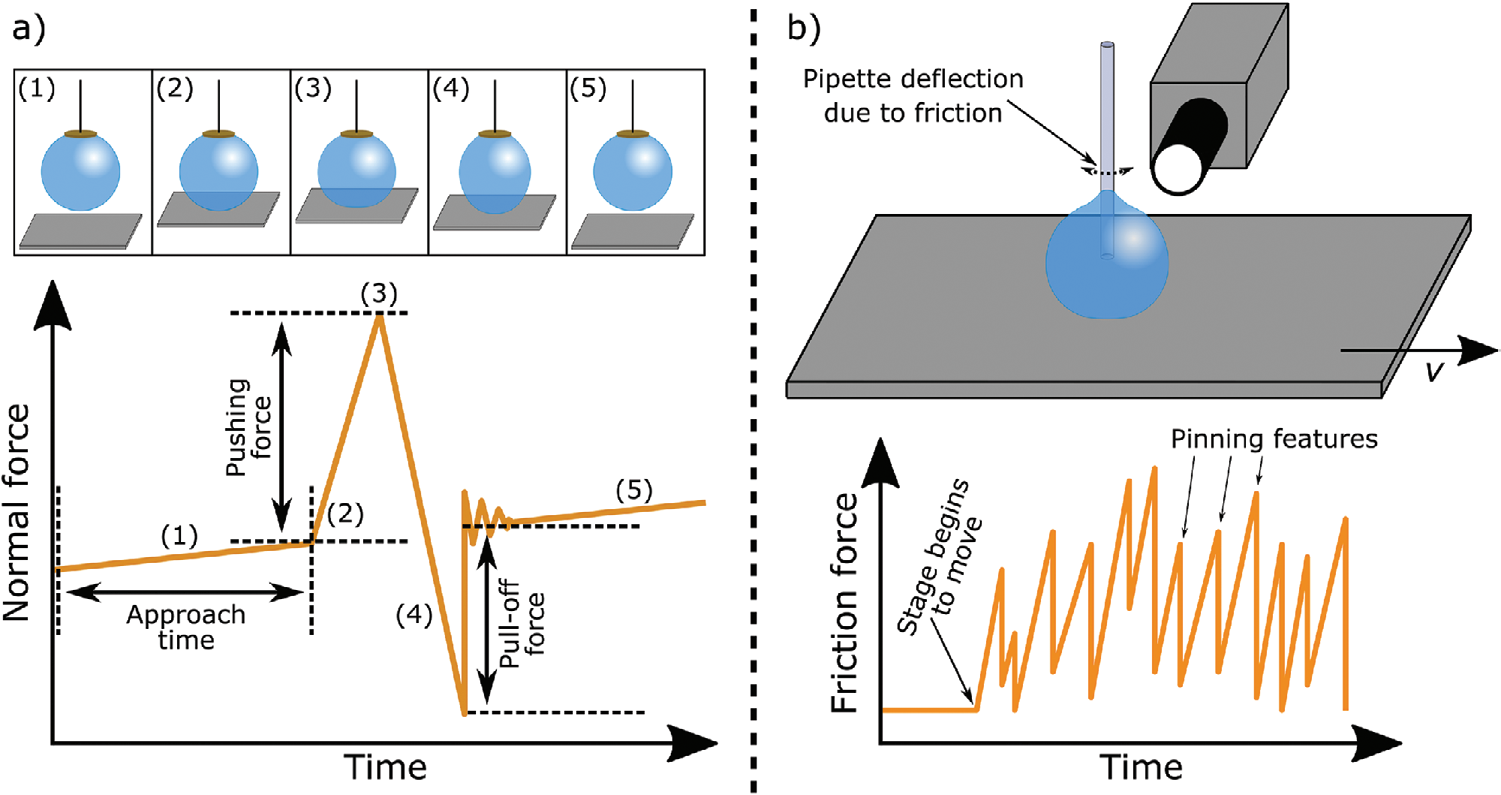
a) Schematic of scanning droplet adhesion microscopy (SDAM) measurement of a super‐repellent surface, including steps (1) through (5) highlighted on a model force curve. 1) Sample is gradually brought closer to the droplet suspended from a sensitive force sensor; evaporation of the droplet causes a small force gradient. 2) Once the surface makes contact with the droplet, the force increases steeply. The approach time will vary point by point on a rough surface, and carries information about the surface topography. 3) The stage approach continues until the pushing force reaches a preset value, and then begins to retract. 4) During retraction, the force decreases until droplet detaches from the surface. 5) The pull‐off force, measured as the difference between the baseline force following the detachment and the global minimum of the force curve, quantifies the local droplet adhesion. b) Schematic of the micropipette force sensor (MFS) droplet friction measurement and a model force–position plot. A droplet injected from a thin capillary is brought into contact with the sample surface, which is then moved laterally at a constant velocity (*v*) while the capillary is stationary. A camera measures the deflection of the capillary in response to the droplet–surface friction. Stick–slip motion of the droplet was observed on the surfaces investigated in this work.

In this work, we quantitatively analyze the wetting variations of stochastic superhydrophobic coatings using two sensitive forced‐based wetting characterization techniques. Scanning droplet adhesion microscopy (SDAM, **Figure** **1**A) is employed to measure the water‐surface interaction in the normal direction.^[^ ^23^ ^]^ Micropipette force sensor (MFS) technique is used to investigate the liquid‐solid friction of water droplets (Figure 1B),^[^ ^24^, ^25^ ^]^ also referred to as the lateral adhesion force. We find that the SDAM technique reveals the non‐uniform nature of the wettability of these coatings, while MFS measurements demonstrate the effects of these inhomogeneities to droplet mobility that shows stick–slip behavior.

In SDAM, droplet–surface adhesion is measured by observing the first contacts of a probe droplet to the surface followed by detachment of the droplet from the surface. Point‐by‐point force measurements are presented as 2D wetting maps.^[^ ^23^, ^26^ ^]^ In this work, mapping the pull‐off forces of water droplets unravels previously unseen variations in the wetting properties of the superhydrophobic coatings on a submillimeter scale. Such subtle features are impossible to probe with CAG, owing to its lack of lateral sensitivity and intrinsic uncertainties on highly repellent surfaces.^[^ ^21^, ^22^ ^]^ Adhesion maps based on measured pull‐off forces can be compared with roughness profiles (see Supporting Information for details) to estimate the interplay between surface topography and the wetting properties.

Flexible glass capillaries have recently been used as force transducers to study the friction of liquid droplets moving on various substrates.^[^ ^25^, ^27^, ^28^, ^29^, ^30^ ^]^ Here we use highly flexible glass capillaries as MFSs with a force sensitivity down to a few nanonewtons^[^ ^25^ ^]^ to quantify the friction of water droplets moving on the superhydrophobic coatings. The measurements reveal complex stick–slip behavior that highlights how contact line motion is affected by the subtle wetting heterogeneities of these surfaces.

## 2 Results and Discussion

**Figure 2:**
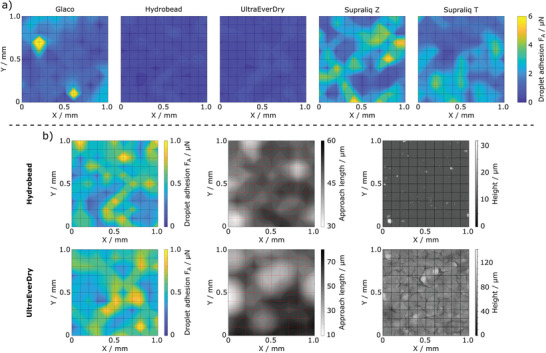
a) Droplet adhesion maps for each coating surface. The data is presented as contour plots, where the 11 × 11 measurement points lie in the mesh intersections. b) Comparison of the droplet adhesion maps (left) for Hydrobead and UltraEverDry coatings based on their approach length (middle), with the respective optical profilometry height maps (right). The SDAM measurement mesh has been superimposed on the profilometry image for clarity. NOTE: Short approach length corresponds to high topography (white regions), while long approach length implies low topography (dark features).

We investigated the wettability of the following stochastic superhydrophobic coatings: Glaco, Hydrobead, UltraEverDry, Supraliq Z, and Supraliq T (see Table S1, Supporting Information, for details). The contact angles (advancing, receding), sliding angles, as well as, the adhesion and lateral (pinning) forces are summarized in **Table** **1**. To map droplet adhesion on the coating surfaces, SDAM measurements on a 1 mm × 1 mm area were done with 100 µm intervals. The resulting droplet adhesion maps on each coating are shown in **Figure** **2**A and Figure S1A, Supporting Information. It can be seen that the adhesion forces are very low and generally remain in the order of a few µN in all measurements, which shows that all of these coatings are highly water‐repellent as compared to, for example, the range of surfaces investigated in our previous work.^[^ ^23^ ^]^ In order to compare measured adhesion forces with macroscopic contact angles, the mean adhesion forces ${\bar{F}}_{A}$ from the obtained force maps presented in Figure 2 were calculated and are listed in Table 1. The standard deviations of the mean adhesion forces ${\bar{F}}_{A}$ reflect the significant regional variations of wettability over the scanned area. Comparing the adhesion forces against the contact angle values shows that the stronger ${\bar{F}}_{A}$ measured on the Supraliq coatings correspond to their slightly lower contact angles, particularly the receding contact angle θ_rec_.

**Table 1:** Summary of all measurement results. From left: Advancing and receding contact angles (θ_adv_ and θ_rec_) with estimated uncertainty due to the positioning of the baseline; sliding angles ($\bar{\alpha }$) with statistical standard deviation; maximum friction forces ${\bar{F}}_{\alpha }\;=\;\rho Vg\;\sin\bar{\alpha }$ computed from the sliding angle measurements, scaled by the mean droplet–surface contact diameter $\bar{D}$ with propagated uncertainty; mean droplet adhesion forces (${\bar{F}}_{A}$) from SDAM sampling of 242 independent force measurements on each surface with standard deviation; mean magnitude of the pinning events (${\bar{F}}_{p}$) and their mean displacement ($\bar{d}$) from the micropipette droplet friction measurements with standard deviations; normalized pinning force ${\bar{F}}_{p}/\bar{L}$ with propagated uncertainty, where $\bar{L}$ is the mean droplet‐substrate contact diameter in the micropipette measurement

Coating	θ_adv_ [°]	θ_rec_ [°]	$\bar{\alpha }$ [°]	${\bar{F}}_{\alpha }/\bar{D}$ [µN mm^−1^]	${\bar{F}}_{A}$ [µN]	${\bar{F}}_{p}$ [nN]	$\bar{d}$ [µm]	${\bar{F}}_{p}/\bar{L}$ [µN mm^−1^]
Glaco	171 ± 10^a)^	169 ± 9	9 ± 4	14 ± 5	1.7 ± 1.3	220 ± 40	80 ± 30	0.8 ± 0.2
Hydrobead	173 ± 5	172 ± 10^a)^	10 ± 4	15 ± 7	0.6 ± 0.6	600 ± 300	160 ± 80	2.1 ± 1.1
UltraEverDry	169 ± 5	168 ± 8	7 ± 7	12 ± 12	0.6 ± 0.3	600 ± 500	300 ± 200	1.5 ± 1.4
Supraliq Z	167 ± 4	163 ± 5	4 ± 3	6 ± 5	3 ± 2	700 ± 200	200 ± 90	3 ± 1
Supraliq T	165 ± 3	163 ± 4	1.8 ± 0.5	3.4 ± 0.9	2.2 ± 1.3	900 ± 300	290 ± 150	3 ± 2

^a)^ Contact angle inaccuracies are based on a model where the droplet baseline is deviated by ± 1 pixel level;^[^ ^31^ ^]^ The reported error is a mean value of the two cases and may thus suggest nonphysical angles beyond 180°; Details of the error analysis are given in Supporting Information.

Based on scanning electron microscopy (SEM) images (Figures S3 and S4, Supporting Information), the coatings generally exhibit distinctly different surface topographies, which is also reflected in the stylus‐based roughness measurement (Table S1, Supporting Information). Figure 2B and Figure S1B, Supporting Information, show SDAM adhesion maps on Hydrobead and UltraEverDry coatings matched with topographic data from the optical profilometer. The matching is based on mapping the approach length inferred from the SDAM force curves, that is, the length of the vertical motion of the sample stage between its initial position and the position where the droplet first contacts the surface, and thus enables tracing the approximate surface topography as experienced by the probe droplet. In comparison with optical profilometry, tall features can still be recognized as distinct minima in the approach length map despite substantial broadening due to the millimeter‐sized droplet.

After the initial contact between the droplet and the surface, the motion of the sample stage continues for a while. This way, we force the droplet into stronger contact with the surface. The droplet will then either sag over the point of initial contact, or shifts laterally. In either case, the droplet may come into contact with additional surface features. During the retraction, the stage gradually moves back, and the moment the droplet snaps off from the surface defines the pull‐off moment. This event is highly dynamic and depends on the microtopography of the droplet–surface contact. For example, even if the droplet only contacts a single, heightened surface feature, the droplet–surface meniscus can rupture either from the top of the feature, or from its side. Thus, it is not surprising that the Pearson correlation coefficients calculated for droplet adhesion and the approach length show relatively weak correlations, and these become even weaker when compared against the topography as measured by optical profilometer (Table S3, Supporting Information). This demonstrates that droplet adhesion is strongly affected by other surface features that contribute to the droplet dynamics and the shape of the droplet–surface contact area, such as surface chemistry.

Contact angle characterization confirms the repellent nature of the coatings, with advancing and receding contact angle values well above 160° on all surfaces. Although carrying out accurate contact angle measurements on such highly repellent and topographically heterogeneous surfaces is very challenging due to, for example, the ambiguity of the baseline position,^[^ ^22^, ^31^ ^]^ the high contact angles are in good agreement with the weak droplet adhesion observed.

Sliding angle characterization was performed to complement the contact angle measurements. In these measurements, clear stick–slip motion of the droplet contact line was evident on all surfaces: Starting from low tilt angles, the contact line may repeatedly jump for a distance along the surface and then become pinned again, only becoming completely released at a much higher tilt angle (Figure S5, Supporting Information). This pinning behavior is attributed to the wetting heterogeneity of the surfaces and leads to a wide statistical spread in the sliding angles α (Table 1), taken as the substrate tilt angle where the droplet completely slides off the area of observation.

**Figure 3:**
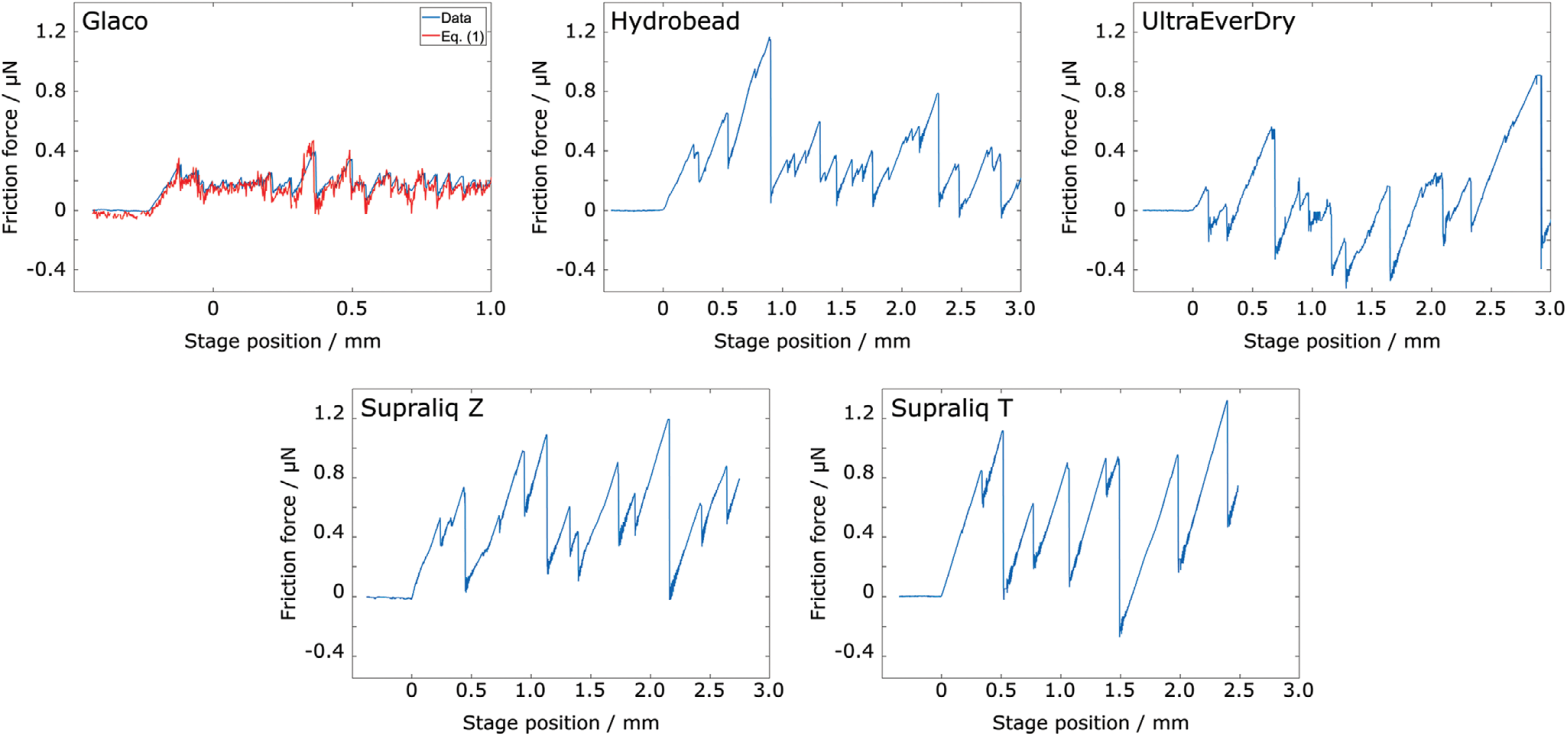
Force–position plots of droplet friction measurements carried out with the micropipette force sensor method. The horizontal axis refers to the motion of the substrate at constant velocity (*v*  =  0.1 mm s^−1^). For Glaco, also the theoretical modelling from Equation (1) is shown.

**Figure 4:**
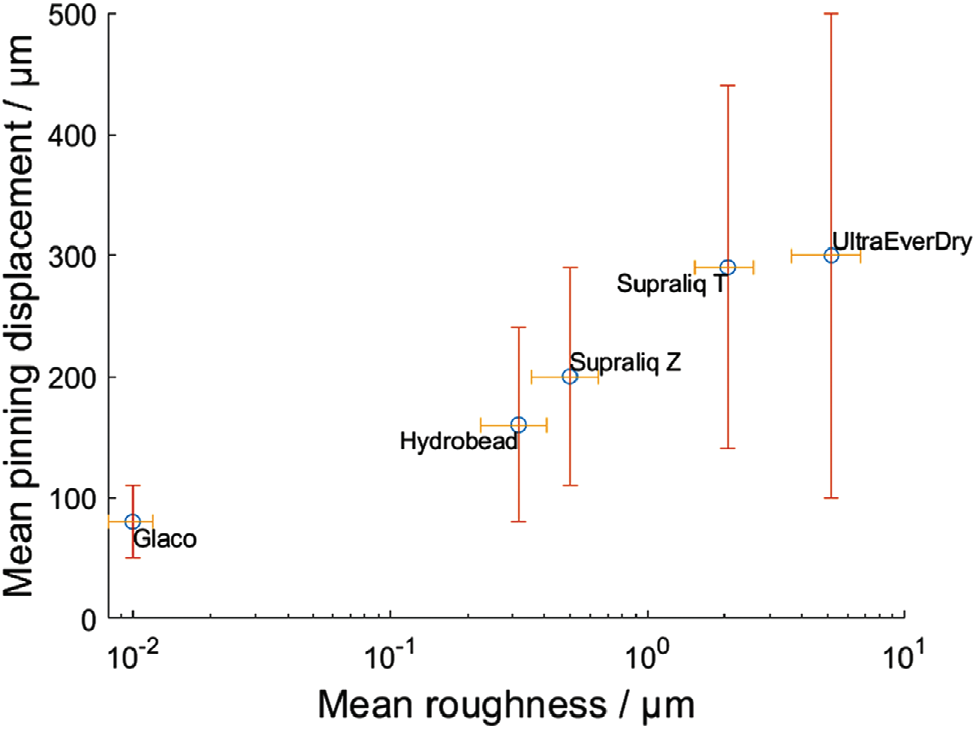
Semi‐log plot of the mean displacement of pinning sites $\bar{d}$ on the coating surfaces (inferred from micropipette droplet friction measurements) against the mean surface roughness ${\bar{R}}_{a}$ from stylus profilometry. It can be seen that mean pinning displacement $\bar{d}$ reflects a semi‐logarithmic relationship with surface roughness.

The stick–slip nature of droplet motion can be accurately probed with MFS friction measurements, where the pinning behavior is analyzed with high spatial resolution (**Figure** **3**). The mean pinning forces ${\bar{F}}_{p}$, that is, the mean magnitude of the stick–slip force maxima, and their mean displacements $\bar{d}$ were inferred from a number of measurements carried out on each surface with droplets of comparable size (Table 1 and Table S2, Supporting Information). Based on these measurements, Glaco shows the most uniform droplet mobility with the lowest mean pinning force and relatively small statistical variations (≈18%) among the studied coatings. In contrast, the ${\bar{F}}_{p}$ values are higher and show more statistical variations (from ≈30% for Supraliq Z to ≈83% for UltraEverDry) on other surfaces. The mean displacement of pinning sites $\bar{d}$ reflects a semi‐logarithmic relationship with the coating roughness, albeit with significant spatial variations: Glaco is smoother than Hydrobead and Supraliq Z, which in turn are smoother than Supraliq T and UltraEverDry (**Figure** **4**). This indicates that the stick–slip motion is dominated by their topographic roughness.

MFS measurements give normalized mean (lateral) pinning force per diameter of the contact region (${\bar{F}}_{p}/\bar{L}$, Table 1) around 0.8–3.1 µN mm^−1^ on all surfaces, which is comparable to a previous report on superhydrophobic silicon nanofilaments (≈2.0 µN mm^−1^).^[^ ^29^ ^]^ Corresponding values calculated for the macroscopic sliding angle measurements (${\bar{F}}_{\alpha }/\bar{D}$ Table 1) are generally an order of magnitude larger than the ones for the MFS measurements. The sliding angle measurement records the strongest individual pinning features encountered by a droplet sliding on the surface along an indeterministic path. This approach ignores any weaker pinning points that coincide along the droplet's path (Figure S5, Supporting Information), and is thus greatly affected by the semirandom nature of the droplet motion. Meanwhile, MFS grants a degree of control over the motion of the droplets, and provides a more deterministic measurement with a detailed view on how surface heterogeneities affect droplet friction.

It is also important to note that individual pinning features seen in the plots of Figure 3 are of similar order of magnitude, meaning that the MFS measurement is not likely to “home” in on the large defects the way the sliding angle measurement with its larger droplet size does. Friction forces measured using sliding angles and MFS therefore demonstrate how wetting properties can be affected by the scale of the measurement. Interestingly, the calculated ${\bar{F}}_{p}/\bar{L}$ and ${\bar{F}}_{\alpha }/\bar{D}$ are nevertheless very comparable on the Supraliq coatings. This may indicate that there are fewer “major” defects on these surfaces for the freely sliding droplet to adhere to, and thus both the measurements are dominated by comparably weaker heterogeneities that densely populate the surfaces.

The results of the MFS measurements can be justified based on the theoretical model of droplet friction force:^[^ ^25^, ^28^, ^32^, ^33^ ^]^

(1) $$F_{\text{fric}}=\frac{24}{\pi^{3}}\gamma L(\cos\theta_{\text{back}}-\cos\theta_{\text{front}})$$

Here, θ_back_ and θ_front_ represent the back and front contact angles, respectively, during the MFS measurement, γ is the surface tension of the drop, and *L* is the contact region diameter. We were able to estimate them on the topographically smoother Glaco and Hydrobead coatings where the baseline is possible to resolve from the side‐view. The comparison of the experimental data and the theoretical model of Equation (1) shows excellent agreement on Glaco (Figure 3). On Hydrobead (Figure S2, Supporting Information), the force values are in order‐of‐magnitude agreement aside from points where the in‐situ contact angle estimation fails momentarily due to the highly dynamic pinning events. These moments are apparent in the in‐situ contact angle data also shown in Figure S2, Supporting Information, which is messy for Hydrobead due to its coarser surface texture. On the other even rougher surfaces, the estimation fails altogether. It must thus be kept in mind that even on the smooth Glaco coating, the theoretical model is subject to substantial uncertainty due to the imprecisions of the optical contact angle measurement.^[^ ^31^ ^]^

The apparent differences between the results of SDAM and MFS also highlight the physical nature of these measurements. The lateral friction force *F*_fric_ depends on the length of the contact line and the difference between the cosines of back and front contact angles (cos θ_back_ − cos θ_front_ in Equation (1)). Aside from superhydrophobic surfaces, high droplet mobility can also be achieved on a variety of slippery surfaces with comparatively low contact angles but very low contact angle hysteresis.^[^ ^30^ ^]^ On the other hand, the normal adhesion force, quantified here as the pull‐off force (*F*_A_), relates to the droplet–surface contact area upon separation of the droplet from the surface, and depends on the receding contact angle θ_rec_ exclusively.^[^ ^23^, ^34^ ^]^ Thus, *F*_A_ only becomes small for very high θ_rec_, that is, for surfaces that are superhydrophobic. In our measurements, this explains how coatings such as UltraEverDy and Hydrobead can show the lowest ${\bar{F}}_{A}$ in SDAM, but comparably high ${\bar{F}}_{p}/\bar{L}$ in MFS.

Our results also demonstrate how surface heterogeneities are reflected differently in SDAM (normal direction) and MFS (lateral direction) measurements. The droplet friction measurement is affected by variations in both surface chemistry and topography that coincide with the path of the droplet. On a highly superhydrophobic but topographically heterogeneous surface, lin‐log relationship between $\bar{d}$ and the surface roughness (Figure 4) suggests that roughness of the coatings appears to dominate the droplet–surface interaction. In SDAM, normal forces are measured in spatially localized manner: Aside from the local receding contact angle θ_rec_, the pull‐off force is affected only by the size and features of the droplet–surface contact area, as demonstrated by earlier measurements carried out on micropillars of varying diameters.^[^ ^23^ ^]^

## 3 Conclusion

This work documents the wetting performance of stochastic superhydrophobic coatings in terms of their wetting heterogeneity, quantified by droplet adhesion (SDAM) mapping and MFS droplet friction measurements. Compared to optical contact angle measurements, SDAM provides substantial insights into the wetting performance of these coatings. While the measured forces are down to µN level, all the investigated coatings still reflect a significant degree of regional heterogeneity for droplet adhesion in sub‐mm scale. Such non‐uniformity of highly superhydrophobic surfaces could not be studied using traditional CAG. Similarly, sliding angles measured on the coating surfaces are only able to predicate information on their wettability on very general level, while MFS enables systematic investigation of the complex pinning behavior that is primarily brought about by sub‐mm topographic variations.

Thus, we establish that sensitive, force‐based characterization techniques can obtain a far more comprehensive picture on the wetting properties of topographically complex surfaces, and demonstrate how wettability depends on the scale of the measurement. In addition, this work highlights how combined SDAM and MFS measurements can decouple the lateral and normal directions of wettability, which is essential for understanding the performance of surfaces in many practical situations (e.g., droplets on tilted vs overturned surfaces).

## 4 Experimental Section

### Sample Preparation

Hydrobead, Glaco, and UltraEverDry coatings were deposited onto 18 mm × 18 mm thermally oxidized silicon wafers with laser‐engraved position reference marks via spray coating according to the instructions provided by the suppliers. To ensure complete evaporation of the solvent, films were cured in ambient conditions for a minimum of 24 h prior to measurements. Supraliq T and Z coatings were received pre‐deposited on the microscopy glass slides, and studied as‐received.

### Sample Characterization

The imaging of the coatings was done by scanning electron microscope. Roughness was measured by stylus profiler. Topography of the Hydrobead and UltraEverDry surfaces was measured using an interferometry‐based optical profilometer.

### Droplet Adhesion Measurements

Droplet adhesion measurements were performed using the SDAM.^[^ ^23^ ^]^ Point‐by‐point scanning were done on 1 mm × 1 mm area with spatial resolution of 100 µm with water droplet probe. The droplet volume was 1.5 μL and kept constant by refilling after each measurement. The scanning was done on each sample at two different areas. Adhesion force was defined as the force when the droplet snaps off from the surface (pull‐off force on Figure 1A, see Supporting Information for more details).

### Droplet Friction Measurements

The friction of water droplets moving on the superhydrophobic samples was measured using the MFS technique.^[^ ^25^ ^]^ Elastic deflection of a highly flexible force‐calibrated glass capillary was used as the force transducer. The deflection (Δ*x*) of the micropipette was analyzed using Matlab^[^ ^24^ ^]^ from side‐view videos showing the bending of the pipette during the measurement, and the friction force was calculated as *F*_fric_ =  *k*_p_Δ*x*. Several spots on each sample were scanned with water droplets of comparable volume (*V* = 0.82 ± 0.05 µL), rendering similar diameters of the contact area $\left(\bar{L}\right)$ between the droplet and the surface (Table S2, Supporting Information). The mean strength of the pinning sites ${\bar{F}}_{p}$ and their mean displacement$\bar{d}$ from one another were inferred from the local maxima of the *F*_fric_ data measured on the different surfaces.

### Contact Angle Measurements

Contact angle measurements were carried out with a commercial contact angle goniometer. Advancing contact angle measurements were done according to the measurement protocol described previously,^[^ ^19^ ^]^ with the droplet volume typically increasing from 4 to 12 μL at a rate of 0.05 µL s^−1^. Receding contact angles in this work were estimated by observing the evaporation of a sessile droplet (8–10 μL in volume) on each surface.

### Error Analysis for Contact Angle Measurements

The errors reported for the contact angle measurements are based on estimated baseline misplacement by 1 pixel. The baseline position is obscured by surface roughness, making the baseline misplacement the dominant source of uncertainty. Estimation of the errors produced by one‐pixel mismatch was calculated using a model from an earlier work.^[^ ^31^ ^]^

### Sliding Angle Measurements

Sliding angles were measured by tilting the contact angle goniometer with the external tilting cradle. The droplet volume in these measurements was 20 μL, and the tilting rate was 5° per minute. Due to the stick–slip nature of the droplet motion on these surfaces, the interpretation of the sliding angle is subject to ambiguity. The sliding angles reported were taken from the tilt angle at which the droplet slid off the image area. Measurements were repeated 5 times on different points of each coating surface.

## Conflict of Interest

All authors are involved in the development of alternative wetting characterization techniques. M.V., Q.Z., and R.H.A.R. are inventors of related patent applications, and together with M.J.H. are considering potential commercialization partly supported by Business Finland and ERC.

## Supporting information

Supporting Information

## Data Availability

The data that support the ﬁndings of this study are available from the corresponding author upon reasonable request.
