# Supplementary material for: Force‐Based Wetting Characterization of Stochastic Superhydrophobic Coatings at Nanonewton Sensitivity
Source: Adv Mater. 2021 Sep 1;33(42):2105130. doi: 10.1002/adma.202105130 (PMC11468561; doi:10.1002/adma.202105130)
Supplement: Supplementary file 1 — Supporting Information [file ADMA-33-2105130-s001.pdf]

© 2021 Wiley-VCH GmbH

ADVANCED MATERIALS

Supporting Information

for *Adv. Mater.,* DOI: 10.1002/adma.202105130

Force-Based Wetting Characterization of Stochastic Superhydrophobic Coatings at Nanonewton Sensitivity

Matti J. Hokkanen, Matilda Backholm, Maja Vuckovac, Quan Zhou, and Robin H. A. Ras**

Copyright 2021, Wiley-VCH GmbH.

Supporting Information

Force-Based Wetting Characterization of Stochastic Superhydrophobic Coatings at Nanonewton Sensitivity

Matti J. Hokkanen, Matilda Backholm, Maja Vuckovac, Quan Zhou, and Robin H. A. Ras**

Contents:

1. Experimental details
2. Supplementary data & figures
    a. Additional SDAM droplet adhesion maps and optical profilometry data
    b. Pearson correlation values for droplet adhesion and topography measurements
    c. Plots of MFS measurements on the coating surfaces
    d. SEM images of the coating surfaces
    e. Stick-slip motion in the sliding angle measurements

1. Experimental details

Superhydrophobic coatings: Hydrobead-T, UltraEverDry (UltraTech International, Inc.) and Glaco Mirror Coat Zero (SOFT99) were purchased as solutions for spray deposition. Glaco and Hydrobead consist of a single-layer treatment and were delivered as ready-to-use products in a retail package equipped with a sprayer. UltraEverDry was delivered as separate bottom and top coat solutions. Supraliq Z and T coatings were supplied by Dr. Pavel Levkin (Karlsruhe Institute of Technology), deposited on microscopy glass slides.

Preparation of the coating samples: Glaco, Hydrobead and UltraEverDry coatings were prepared on 18 mm x 18 mm silicon wafer pieces with a thermally grown oxide layer. 4-inch silicon wafers were patterned and cut into pieces with a laser engraving tool (Cencorp 300 LM). The reference pattern prepared on each sample consisted of 90 degree wedges, each pair defining the opposite corners of a 1.5 mm x 1.5 mm measurement site. Nine such sites were patterned on each sample, arranged into a 3 x 3 matrix separated by ~ 4 mm from each other. Prior to deposition of the coatings, the silicon substrates were cleaned thoroughly in a three-step cleaning process: 1) scrubbing with cotton sticks in acetone, followed by rinsing in isopropyl alcohol; 2) 10-minute ultrasonication in a dilute solution of Deconex 11 (a mild alkaline cleaning solution), followed by rinsing in MilliQ water; 3) 5-minute processing in oxygen plasma to homogenize surface chemistry. The three products were deposited by spray coating. Glaco and Hydrobead were sprayed using the sprayer integrated in the retail package. Bottom and top layers of the UltraEverDry were sprayed using separate disposable Preval sprayers, with 20 minutes of curing time allowed between the layers.

All surfaces were sprayed horizontally, and then lifted vertically for curing for 20 minutes. The samples were then stored in horizontally, and a minimum of 24 hours of additional curing was allowed prior to measurements. Following the deposition of the coating, the reference patterns are visible both in the top-view microscope of the SDAM and the optical profilometer image, and thus enable localization of the measurements.

Table S1. Summary of the coating samples investigated in this work, and their roughness values (mean roughness $\bar{R}_a$ and RMS roughness $\bar{R}_q$ with their respective standard deviations) as measured with a stylus profilometer. $\bar{R}_a$ and $\bar{R}_q$ represent mean values from five independent line scans 1 mm in length.

Coating name	Substrate	$\bar{R}_a$ [µm]	$\bar{R}_q$ [µm]
Glaco Mirror Coat Zero	Si/SiOx	0.010 ± 0.002	0.013 ± 0.003
Hydrobead Standard	Si/SiOx	0.32 ± 0.10	0.52 ± 0.14
UltraEverDry	Si/SiOx	5.2 ± 1.6	6.5 ± 2.1
Supraliq Z	Glass	0.5 ± 0.15	0.69 ± 0.26
Supraliq T	Glass	2.1 ± 0.6	2.7 ± 0.8

Sample characterization:

- **Scanning electron microscopy:** Zeiss Sigma VP (high vacuum mode). Coatings prepared on silicon wafers could be imaged readily. For the Supraliq coatings on glass slides, a 2 nm film of iridium was first applied by sputtering. SEM images were inspected using ImageJ image processing toolkit; the contrast and brightness of some of the images were adjusted manually to improve clarity.
- **Stylus profilometry:** Bruker Dektak XT, equipped with a LIS 3 type B stylus with a 2 µm nominal tip radius. Scan length was set to 1000 µm and duration to 30 s, resulting in a point resolution of ~ 0.11 µm point^{-1}. Stylus force of 29.4 mN (3 mg) was applied. The roughness values were obtained by analyzing the line scans in Gwyddion. The mean values of 5 scans at different positions on each coating are reported.
- **Optical profilometry:** Bruker Contour GT-X. Imaging was done with a 20x objective lens. The automated process provided in the Bruker Vision64 control software was used to record a set of individual, partially overlapping images and stitch them together to make up a 1.5 mm x 1.5 mm composite image of the measurement site defined by the reference marks. Scan length was selected to accommodate the height variations on each coating.

- **Contact angles & sliding angles:** Contact angle goniometer (Biolin Attension Theta) equipped with an external tilting cradle. Advancing and receding contact angles were obtained using the protocol described by Huhtamäki *et al*.[1]

Description of the droplet adhesion (SDAM) measurements: Technical details of the SDAM measurements have been described in a previous work.[2] In these measurements, a water droplet of 1.5 μL was first injected onto the SDAM force sensor from an external dispenser. On each measurement point, the sample is gradually brought into contact with the droplet by approaching with the vertical sample stage at a constant approach velocity (here, 5 μm s^{-1}), and then retracted until the wetting meniscus ruptures (retraction velocity 10 μm s^{-1}). The force sensor records the droplet-surface interaction force continuously during each measurement. The droplet is refilled to the original volume prior to each measurement, to compensate for its evaporation. To position the SDAM measurement in relation to the reference grid, a separate top-view microscope was used to determine the origin point of the map with the help of the prefabricated reference marks on the silicon substrates. The maximum lateral mapping resolution of the technique is chiefly limited by the droplet-surface contact area that depends on the wettability of the surface and the diameter of the measurement droplet. The contact area varies during a typical SDAM measurement as the droplet is first pushed against the surface and then retracts, but its diameter can be coarsely estimated from side-view imaging; with the 1.5 μL measurement droplet, the diameter is in the order of a few hundred μm on superhydrophobic surfaces.

Explanation of the SDAM force curve sampling: On superhydrophobic surfaces with contact angles above 160°, the snap-in forces typically fall below the detection limit of the force sensor. In SDAM measurements carried out on such surfaces, the vertical stage can be set to approach the force sensor at a fixed velocity until a pre-determined peak force value is reached. In the context of these coatings, there is an additional concern brought about by their

topographic variations: due to the roughness of the coating film, the approach time required to bring the droplet into contact with the surface can vary for different measurement points. This has two important consequences. First, the approach time of the stage carries information about the local surface topography at each measurement point. In this work, this parameter converted to approach length is mapped from each force curve, along with the pull-off force, and used to link the SDAM wetting map with surface topography measurement made using the optical profilometer. Second, since variations in the approach length result in small differences in the droplet volume (due to its gradual evaporation) at the beginning of the contact, it is necessary to control the relative force with which the droplet is pushed against the surface (Figure 1A), rather than the absolute peak force alone. However, due to the response time of the measurement system, the practical pushing force applied will always be greater than the defined value. Furthermore, it will also show slight variations point-to-point due to the non-deterministic nature of the droplet-surface contact. In these measurements, the target pushing force of 0.4 μN in all measurements translated to an applied pushing force of roughly 0.55 ± 0.1 μN as determined in the post-analysis, with a standard deviation of less than 0.05 μN within each individual map of 11 x 11 measurement points.

Analysis of the SDAM force curves: In this work, droplet adhesion is defined as the pull-off force, measured as the difference between the global minimum of the force curve and the corresponding baseline values. The analysis and plotting of the droplet adhesion maps were carried out using a custom MATLAB analysis script.

Matching of SDAM and optical profilometry data: The topographic surface profiles obtained from the optical profilometry on Hydrobead and UltraEverDry coatings were matched with SDAM wetting maps by using the approach length parameter mapped from the SDAM force curves. Approach length is directly proportional to the approach time it takes for the droplet to contact the sample surface during the stage approach with a constant approach velocity

(5 μm s^{-1}). Therefore, it varies point-by-point on a rough surface where it reflects the inverse of the surface topography. Matching was done by first overlaying the topography data with the approach length map in ImageJ, and manually adjusting for the best apparent fit between maxima and minima. After matching, a SDAM measurement grid was then drawn onto the topography image to allow comparison against the droplet adhesion data.

The micropipette force sensor technique: In the micropipette force sensor measurements, the sample was placed on a motorized stage and the micropipette was connected to a syringe filled with MilliQ water and mounted in a vertical direction above the sample. A straight micropipette (2.5 cm long and 30 μm outer diameter) was pulled from a hollow borosilicate glass capillary (outer/inner diameter 1/0.75 mm, TW100-6, World Precision Instruments, USA) using a micropipette puller (PN-31, Narishige, Japan), and cut using a microforge (MF-900, Narishige, Japan). Using the syringe, a water droplet was pushed out through the end of the micropipette until it was big enough (typical volume ~ 1 μL) to slide down along the pipette tip onto the sample. The droplet and micropipette were imaged at a frame rate of 50 fps using a Phantom Miro 310 camera (Vision Research Inc., USA) equipped with a macro lens (Canon MP-E 65 mm f/2.8 1−5× Macro Photo) at its highest magnification (∼ 4 μm pxl^{-1}). To damp out external disturbances, the setup rested on an active vibration isolation table (Accurion Halcyonics, i4 Series) placed on an optical desk, and all measurements were made in a closed room with ventilation turned off. In the experiment, the camera was triggered about 4 s before the motor was started in order to record the zero-force level of the micropipette. The sample stage was accelerated at $a = 4.0$ mm s^{-2} to a constant velocity of $v = 0.1$ mm s^{-1} and moved for 20–30 s. This measurement was repeated four to seven times on each coating (Table S2). Prior to the measurements, the pipette was calibrated using the procedure described in detail in a preceding work,[3] rendering a spring constant $k_p = 5.6\ \pm$

0.1 nN μm^{-1}, where the error corresponds to the standard deviation of four calibration experiments.

Analysis of the droplet friction measurements: In the analysis, each local force maximum in the F_{lat} measurement is treated as a pinning point, with a pinning force of F_p corresponding to its magnitude (relative to the zero-level). The mean pinning force $\overline{F_p}$ reported in Table 1 is the pooled mean of *N* measurements carried out on each coating, and the reported uncertainty is the statistical standard deviation associated with the mean values of the different measurements. The mean distance of the force maxima, $\bar{d}$, is resolved similarly for each coating, with the statistical standard deviation from the different measurements represented as uncertainty in Table 1. Thus, $\bar{d}^{-1}$ can be regarded as the mean density of the pinning points on each coating.

It is important to note that in a dynamic measurement, weak pinning features are masked from view as the droplet can jump over them following its release from a stronger neighbor. Thus $\bar{F}_p$ and $\bar{d}$ tend to be elevated on surfaces where strong individual pinning events take place. In the force curves presented in Figure 3, it can also be seen that sometimes the force momentarily falls below the zero-level following a large pinning feature. This artefact is likely a result of the droplet sliding down along a negative slope after being released from a pinned state, which can cause the pipette to "jump ahead" of its equilibrium position.

Table S2. Details of the MFS measurements: supplementary to Table 1, including the mean diameter of the contact area $\bar{L}$ and the number of measurements *N* carried out on each coating.

Coating	$\bar{F}_p$ [nN]	$\bar{d}$ [μm]	$\bar{L}$ [μm]	$\bar{F}_p/\bar{L}$ [μN mm^{-1}]	*N*
Glaco	220 ± 40	80 ± 30	260 ± 30	0.8 ± 0.2	4
Hydrobead	600 ± 300	160 ± 80	290 ± 40	2.1 ± 1.1	4
UltraEverDry	600 ± 500	300 ± 200	390 ± 80	1.5 ± 1.4	7
Supraliq Z	700 ± 200	200 ± 90	250 ± 40	3 ± 1	5
Supraliq T	900 ± 300	290 ± 150	290 ± 50	3 ± 2	5

This behavior, which is most profound on rough substrates such as UltraEverDry with very strong pinning sites, results in further masking of weak pinning events. $\bar{F}_p$ and $\bar{d}$ values should therefore only be regarded as qualitative descriptors of the pinning behavior on these surfaces. For the normalized pinning force $\bar{F}_p/\bar{L}$, the diameter of the contact area L was estimated from the side-view videos for each coating (Table S2).

Contact angle goniometry: Contact angle measurements on these coatings were challenging for several reasons. First, the highly repellent nature of the surface always results in large uncertainties for advancing and receding contact angle values in an individual measurement due to ambiguity of the baseline position.[4] This is an intrinsic feature of the optical method, which becomes particularly pressing on surfaces such as stochastic commercial coatings – these also feature visible microscale roughness that further obstructs the base region of the droplet in the side-view image. Second, non-uniform wetting behavior across the surface results in the contact line moving in jumps, making it ambiguous when the advancing and receding contact angles are reached. Finally, due to the high mobility of the droplet on these extremely repellent surfaces, it is very difficult to insert the injection needle inside the droplet for the receding angle measurement. Proper placement of the needle tip inside – and in the center of – the droplet is important: if the needle merely touches the top of the droplet in the beginning of the measurement, it will exert a pulling force onto the shrinking droplet that will result in anomalously high receding angles. Unfortunately, our attempt to bring the needle down into the droplet on such a slippery surface always caused the droplet to escape the needle, often pinning onto its side instead. In such configuration, the needle will withdraw air, rather than the liquid. Advancing contact angle measurements were carried out by following the recommended protocol,[1] by increasing the volume of the measurement droplet at a drop rate of 0.05 μl s^{-1} from 4 μl to 12 μl in a typical measurement. From the plot of contact angle vs. droplet volume on this range, a region corresponding to a volume increase of at least 0.5

μl was selected where the measured contact angle was closest to a constant value, and the mean contact angle from that range was taken as the advancing contact angle. The same approach could not be utilized for the receding contact angle measurement, because it was impossible to insert the needle appropriately inside the droplet on the highly repellent surfaces. The receding contact angles were estimated in the absence of the needle, by observing spontaneous evaporation of a sessile droplet carefully deposited on the sample surface. The starting volume of the evaporating droplet was between 8 and 10 μl on each surface. Similar to the advancing case, a mean contact angle corresponding to a volume interval of at least 0.5 μl was taken as the receding contact angle, at the region of the contact angle vs. droplet volume plot where the contact angle varied the least. In all measurements, the droplet baseline was determined manually at the perceived vertical position of the sample surface.

Error analysis for contact angle measurements: In the analysis of the contact angle measurements, the uncertainty associated with the baseline position is assumed to be the dominant error source. The error estimates given in Table 1 are based on a calculation from an analytical model for a one-pixel mismatch of the baseline from the true position.[4,5] In this analysis, droplet profiles were first derived from the Young-Laplace equation for a given droplet volume and contact angle, chosen as a representative measurement on each surface. Then, the Young-Laplace curve was scaled to pixels according to camera resolution and discretized in the z-direction to have one data point in each pixel. After this, contact angle values corresponding to baselines shifted both one pixel up and down from the nominal position were calculated. These yielded two error estimates when compared against the measured contact angle value, and the final error estimate was taken as their mean. This analysis was repeated for both the start and end points of the chosen volume interval, and from these the larger value was reported as the error in Table 1. The same analysis was

carried out for advancing and receding contact angles. Complete details of the modelling approach have been reported elsewhere.[5] It should be noted that while the uncertainties derived in this way are large, the assumption of a single-pixel mismatch of the baseline is only realistic for smooth surfaces. On very rough coatings such as UltraEverDry, where the sample surface level is difficult to resolve in contact angle goniometry, the error is likely to be even higher. This supports our assumption that the baseline position is the dominant error source in the optical contact angle measurements on these coatings, and further underlines the problematic nature of these measurements on such surfaces.

2. Supplementary figures & data

2a. Additional SDAM adhesion maps and optical profilometry data

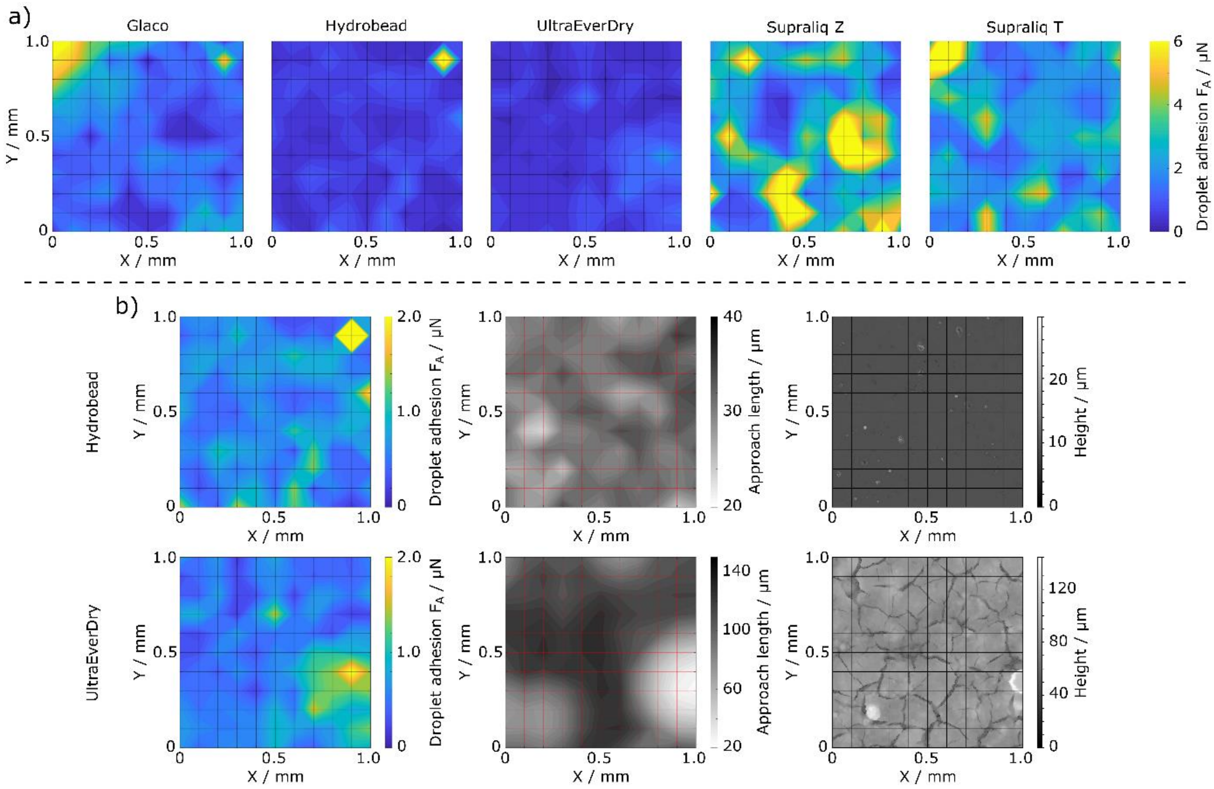

Figure S1. (a) Additional droplet adhesion maps for each coating surface. The data is presented as contour plots, where the 11 x 11 measurement points lie in the mesh intersections. (b) Comparison of the droplet adhesion maps (left) for Hydrobead and UltraEverDry coatings based on their approach length (middle), with the respective optical profilometry height maps (right). The SDAM measurement mesh has been superimposed on the profilometry image for clarity. NOTE: short approach length corresponds to high topography (white regions), while long approach length implies low topography (dark features).

2b. Pearson correlation values for droplet adhesion and topography measurements

In order to investigate the possible correlation between surface topography and wettability, two sets of Pearson correlation coefficients (Table S3) were calculated. First, for each measurement, we investigated the correlation between the droplet adhesion and the associated approach length as measured by the pull-off force SDAM sampling. Second, we also calculated the correlation values between the optical profilometry mapping and the pull-off force for the Hydrobead and UltraEverDry surfaces.

For the latter case, the 2D profilometry data (consisting of ~1000 x 1000 data points) was first binned into rectangular data blocks around the SDAM measurement points (121 x 121 data points). We then calculated the Pearson values by two methods: 'mean', where the mean value of each topography data block was used in the calculation, and 'max', where the maximum value was used. In Table S3, it can be seen that the 'max' method results in stronger correlation magnitudes. This is reasonable, since the large curvature of the SDAM measurement droplet means that it preferentially touches the tallest features of the surface first.

Based on the values shown in Table S3, it is evident that the correlations between the topographic values and wettability are generally weak. The wetting heterogeneities observed in the SDAM measurements do not thus generally appear to originate from topographic variations, as characterized either in-situ or via separate optical measurement. The heterogeneities must thus be caused by either variations of the surface chemistry, or such details of the surface microtopography that could not be resolved by the optical profilometer.

Table S3. Pearson correlation values calculated for the surface wettability and topography using different methods. The data for external topographic measurement is available only for surfaces that could be investigated using the optical profilometer. Negative sign implies negative correlation between topography and wettability, e.g. tall surface features are associated with lower pull-off force in the wetting measurement. It is important to note that the approach length of the SDAM maps the inverse of the topography as seen by the measurement, and thus the signs of the values in the third column have been inverted.

Coating	Measurement area	Approach length – Pull-off force	Topography – Pull-off force [mean]	Topography – Pull-off force [max]
Glaco	1	0,02	-	-
	2	-0,24	-	-
Hydrobead	1	-0,40	0,03	-0,21
	2	-0,23	-0,03	-0,17
UltraEverDry	1	0,54	0,19	0,37
	2	-0,22	-0,12	-0,11
Supraliq Z	1	-0,54	-	-
	2	-0,50	-	-
Supraliq T	1	-0,37	-	-
	2	-0,40	-	-

2c. Plots of MFS measurements on the coating surfaces

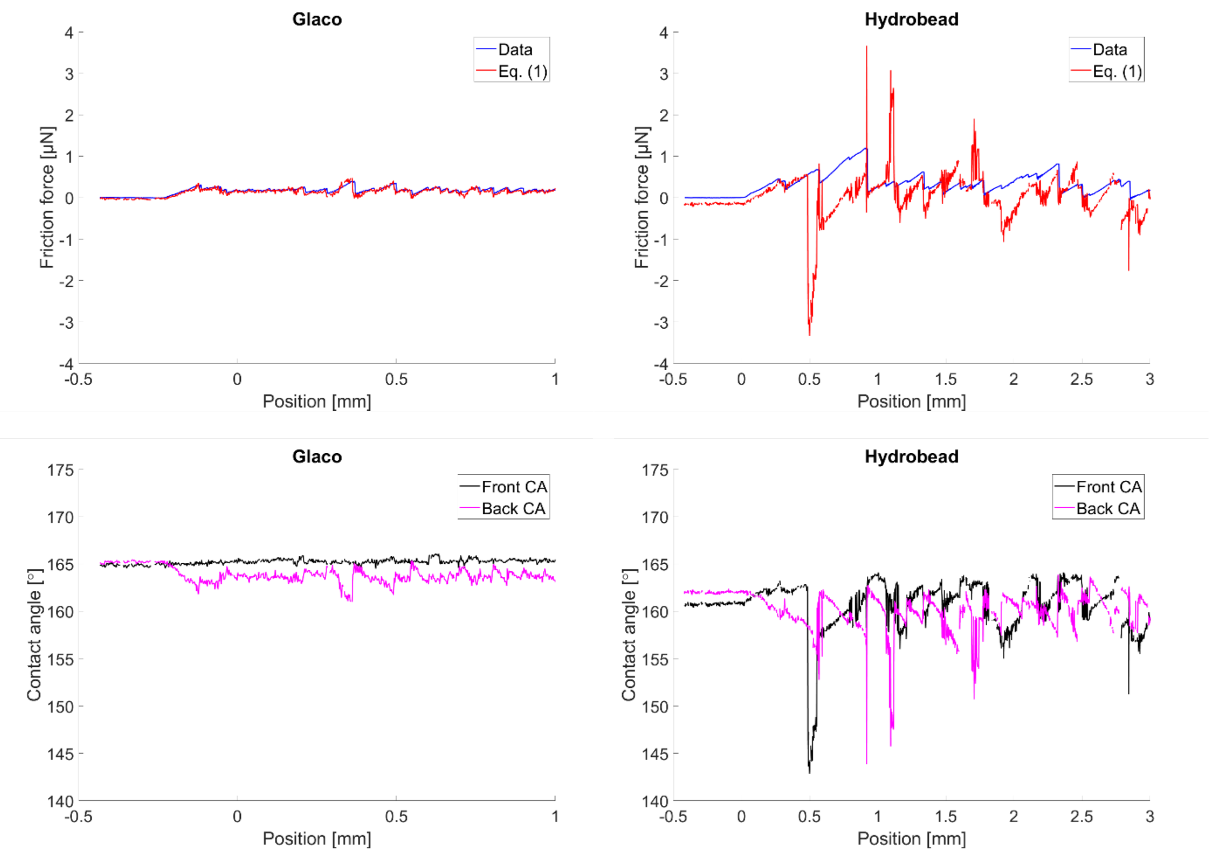

Figure S2. Comparison of the MFS friction force measurement with the theoretical model on Glaco and Hydrobead surfaces. Lower plots show the in-situ front and back contact angles, inferred from the side-view camera images. The contact angles cannot be measured accurately on the other coatings due to the greater surface roughness that completely obscures the droplet baseline.

2d. SEM images of the coating surfaces

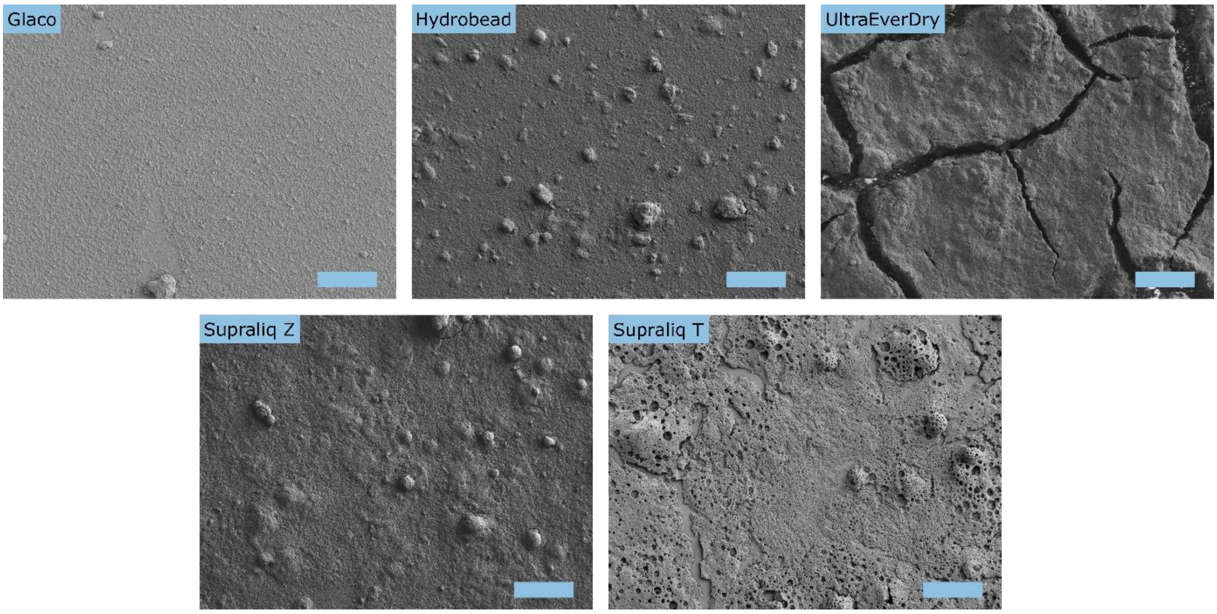

Figure S3: SEM images of the investigated coating surfaces at 300 X magnification; scale bar 50 μm.

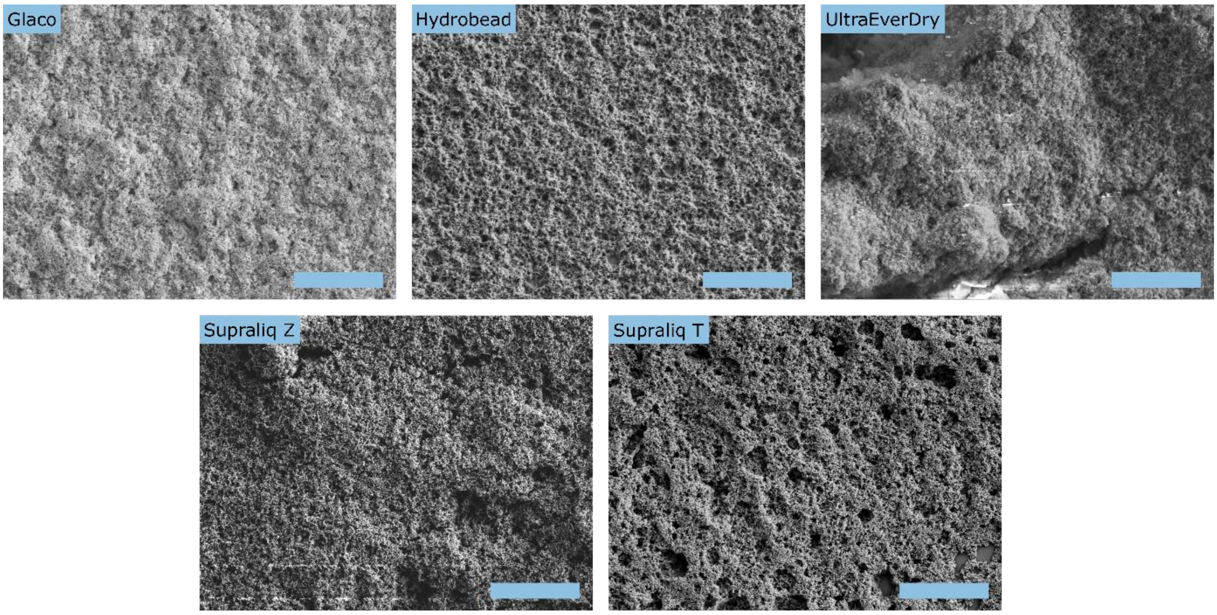

Figure S4: SEM images of the investigated coating surfaces at 5000 X magnification; scale bar 5 μm.

2e. Stick-slip motion in the sliding angle measurements

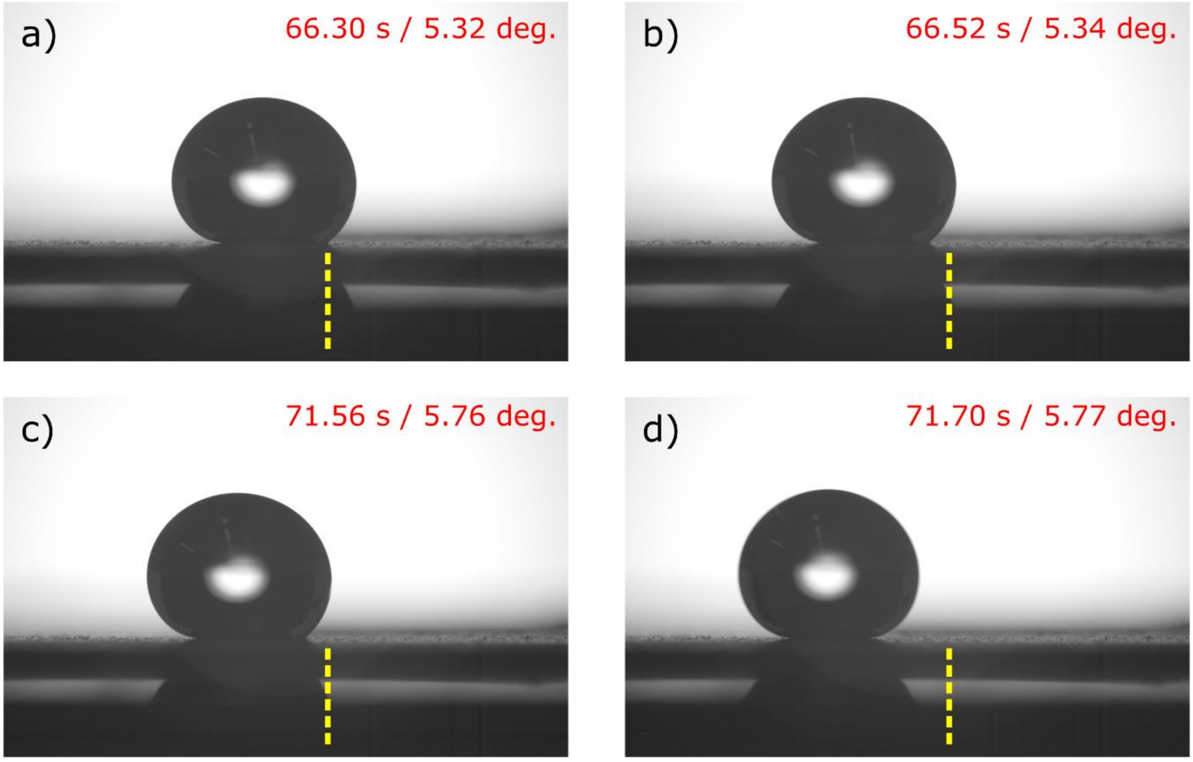

Figure S5: Example of the stick-slip behavior observed in the sliding angle measurements (Hydrobead coating). The droplet is momentarily pinned due to the surface irregularities (a), and then suddenly jumps forward for a small distance (b) over a brief time interval. The droplet then becomes pinned again for an extended period of time (c) until the final release (d) at which point it slides off the surface. Similar behavior can be observed on most surfaces. The droplet volume is ~20 μl, and the tilting rate is 5° per minute (counter-clockwise). The red text shows the time stamp and the cradle angle on each frame. The yellow dashed line is a fixed position reference.

References

[1] T. Huhtamäki, X. Tian, J. T. Korhonen, R. H. A. Ras, *Nat. Protoc.* **2018**, *13*, 1521.

[2] V. Liimatainen, M. Vuckovac, V. Jokinen, V. Sariola, M. J. Hokkanen, Q. Zhou, R. H. A. Ras, *Nat. Commun.* **2017**, *8*, 1798.

[3] M. Backholm, O. Bäumchen, *Nat. Protoc.* **2019**, *14*, 594.

[4] K. Liu, M. Vuckovac, M. Latikka, T. Huhtamäki, R. H. A. Ras, *Science* **2019**, *363*, 1147.

[5] M. Vuckovac, M. Latikka, K. Liu, T. Huhtamäki, R. H. A. A. Ras, *Soft Matter* **2019**, *15*, 7089.
